# Global research trends on psychological well‑being of children in foster care homes from 2003 to 2023: a bibliometric analysis using Scopus database

**DOI:** 10.3389/fpsyg.2024.1397624

**Published:** 2024-08-29

**Authors:** Sandhiya Priyadarshini D, Tony P. Jose

**Affiliations:** Department of Social Sciences, School of Social Sciences and Languages, Vellore Institute of Technology, Vellore, India

**Keywords:** psychological well‑being, foster care homes, orphans, bibliometric analysis, Scopus database, VOSviewer

## Abstract

**Purpose:**

The study focuses on contemporary trends in the psychological well‑being of foster children residing in care homes over the past two decades. Moreover, it aims to predict future research directions by analyzing hotspots, offering valuable references for academics engaged in further studies in this field.

**Methods:**

A quantitative analysis was conducted on nine hundred and twelve documents, including research papers and reviews, from 2003 to 2023. These publications focused on the psychological well‑being of foster children and were incorporated from the Scopus database. For visual analysis, we utilized the bibliometric analytical tool VOSviewer to generate a map of information on the authors, journals, organizations, nations, citations, and keywords. We also employed Microsoft Excel tables to record the essential details.

**Results:**

The psychological well‑being of children and adolescents residing in foster homes is an emerging area of research. The findings show that there has been an ongoing rise in relevant research publications over time. The United States (416) and Harvard Medical School (56) were the most active countries and organizations in this study. *AIDS Care: Psychological and Socio-Medical Aspects of AIDS/HIV* (91 articles) and *Vulnerable Children and Youth Studies* (86 articles) are two prominent journals, while the *Journal of Child Psychology and Psychiatry* had the most co-citations (630). Nelson (52 publications) and Zeanah (50 publications) are the top two leading authors based on citation counts. Institutional care, orphans, *HIV/AIDS* orphans, psychological well‑being, and mental health, resilience are popular research keywords in this study.

**Conclusion:**

This study indicates the prevailing interest in the specified domains over the past two decades. Our findings primarily indicate that addressing mental health concerns, along with understanding the needs of children in foster care homes, can enhance their psychological well‑being. Developing effective interventions to enhance the psychological well‑being of children in foster care is bound to have a profound effect on them and will serve as a key focus for future research in this field.

## 1 Introduction

Foster care provides a safety net for children facing challenging circumstances, ranging from poverty and parental separation to maltreatment and the loss of a parent (Suzuki and Tomoda, 2015). While a significant proportion of children in foster care are not orphans in the traditional sense, with 50–90% having a surviving parent (Bunkers et al., 2014), they often experience disruptions in their biological families due to factors like poverty, parental illness, abuse, or inadequate support networks. Globally, an estimated 140 million children were living in foster care as of 2017 (Petrowski et al., 2017; UNICEF, 2017). Data from India in 2022 indicated 1,53,827 children enrolled in foster care, with the majority having one surviving parent (Ministry of Women and Child Development, 2022). Regardless of their specific circumstances, these children often face significant challenges in self-identity development, social–emotional well‑being, and overall life adjustment (Madihie and Noah, 2013). The experience of separation from biological families, often compounded by histories of neglect or trauma, can profoundly impact the emotional, behavioral, and cognitive development of children in foster care (Erol et al., 2010; Kaur et al., 2018). This heightened exposure to adversity underscores the crucial need for research focused on enhancing the well‑being of these vulnerable children.

Given the adversity these children face, psychological well‑being is paramount. It is a crucial aspect of overall health and quality of life, encompassing emotional, mental, and social health. Psychological well‑being is not merely the absence of mental illness; it represents a multifaceted concept encompassing positive aspects of mental health. Researchers have conceptualized and identified key components of psychological well‑being, including self-acceptance, autonomy, personal growth, environmental mastery, positive relationships with others, and a sense of purpose or meaning in life (Ryff, 1989; Kúld et al., 2021). These components highlight the importance of cultivating a positive self-perception, nurturing meaningful relationships, and developing the ability to navigate life’s challenges. It fosters a sense of contentment with oneself and one’s connections with others (Dey and Daliya, 2019). For children in foster care, promoting psychological well‑being provides a supportive and nurturing environment. This means prioritizing their emotional needs, fostering positive relationships, and cultivating a sense of purpose and personal growth. Addressing these key dimensions can empower these children to develop resilience, navigate challenges, and ultimately thrive. However, enhancing the well‑being of children in foster care is a complex endeavor that presents significant challenges, demanding rigorous and nuanced research.

While psychological well‑being is a significant area of study, frequently measured in large-scale social surveys, research specifically focusing on the psychological well‑being of children in foster care, particularly using bibliometric analysis, remains limited. This paper addresses this gap by employing VOSviewer, a bibliometric analysis software, to visually analyze two decades of research on the psychological well‑being of children in foster care, drawing upon the published literature available in the Scopus database. Unlike traditional reviews, bibliometric analysis utilizes quantitative methods and visualization tools to map the relationships between research articles, authors, periodicals, and institutions, offering a clearer understanding of the field’s evolution and highlighting future research directions. Despite the previous synthesis, to the best of our knowledge, no studies have attempted to analyze the trends in the psychological well‑being of children in foster care research using bibliometric analytic tools. Hence the study aims to fill this research gap.

### 1.1 Aim of the study

This bibliometric study aims to provide a comprehensive overview of the research landscape concerning the psychological well‑being of children in foster care. By examining the annual distribution of publications, the study reveals the growth and evolution of this critical research area. Furthermore, the study identifies and acknowledges significant contributions from various countries, organizations, and influential authors. The analysis also identifies impactful journals disseminating this research and explores prominent research topics and emerging trends within the field.

## 2 Methodology

### 2.1 Data source

Bibliometrics provides a powerful lens for examining scientific literature, offering both quantitative and qualitative insights into publication patterns, influential authors, leading institutions, and emerging trends within a research field (Zhou et al., 2021). This study employed a bibliometric approach to map two decades of research on the psychological well‑being of children in foster care homes. We specifically utilized SCOPUS, a comprehensive bibliographic database launched by Elsevier in 2004, as our primary data source. Scopus stands out for its extensive coverage of peer-reviewed literature across diverse disciplines, encompassing science, social science, medicine, technology, arts, and humanities. It offers researchers a robust suite of tools for tracking citations, analyzing research trends, and visualizing author and institutional networks. Its features, such as h-index calculations and citation analysis, allow for the identification of impactful publications and influential works within a field. The database’s extensive coverage, encompassing over 28,000 titles from more than 7,000 international publishers, makes it more valuable resource for researchers seeking a holistic understanding of the research landscape. Scopus provides essential publication details, including journal name, year of publication, document type, author affiliations, citations, and h-index, enabling comprehensive analysis and meaningful insights (Sweileh, 2018).

### 2.2 Data collection

**Figure 1:**
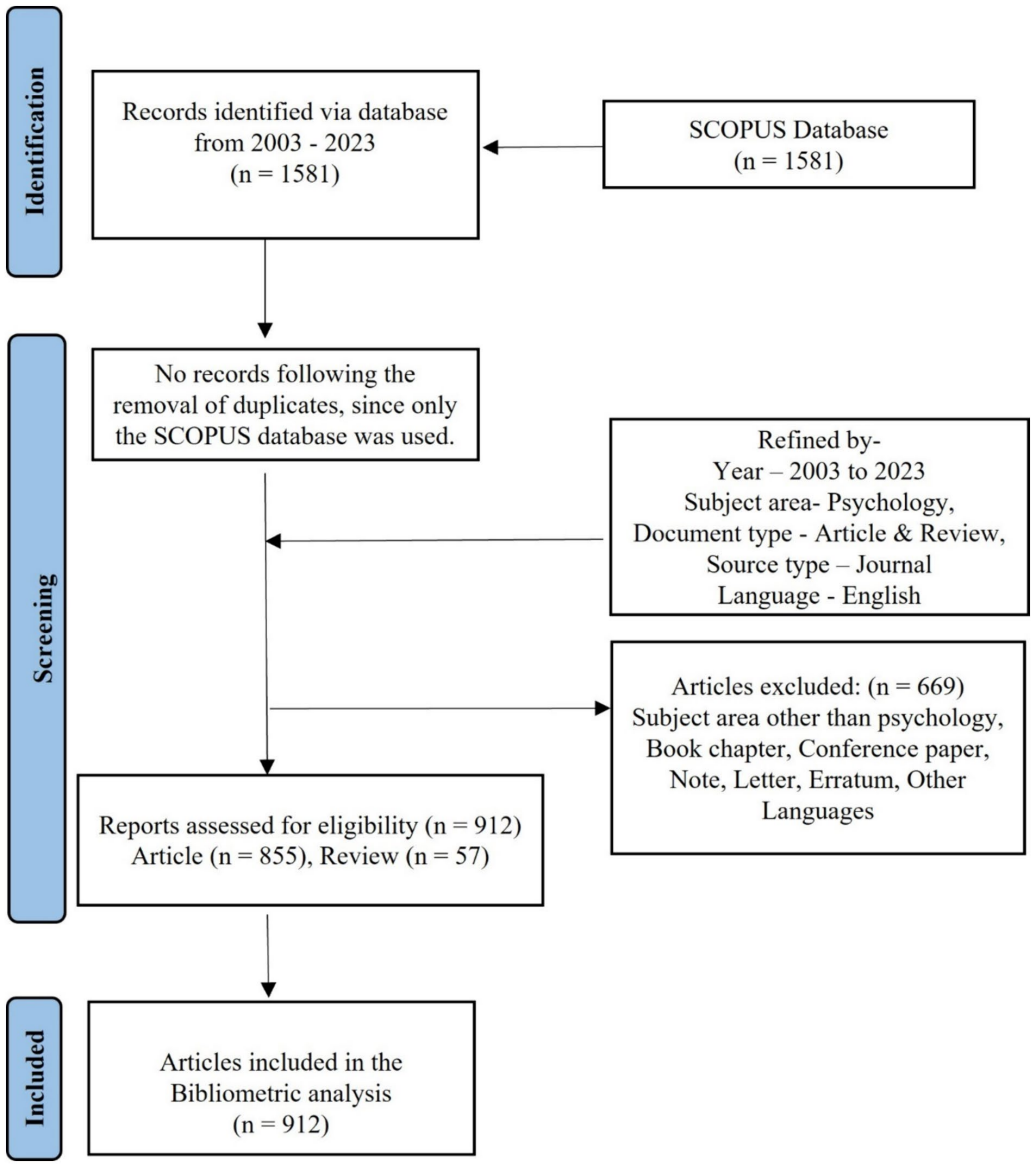
PRISMA flow chart of the selection process.

Keyword selection is crucial in bibliometric analysis, directly impacting the findings and outcomes. This study employed a rigorous search strategy to minimize false positives, focusing on the title/abstract fields within the Scopus database. The search string, executed on January 5th, 2024, was constructed as follows: [TITLE-ABS-KEY (“Psychological Well-being” OR “Well-being”) AND (“Children” OR “Adolescents” OR “Foster Care Homes” OR “Residential Care” OR “Institutionalized Children” OR “Orphan” OR “Semi-Orphan”) AND PUBYEAR >2002 AND PUBYEAR <2024 AND [LIMIT-TO (SUBJAREA, “PSYC”)] AND [LIMIT-TO (DOCTYPE, “ar”) OR LIMIT-TO (DOCTYPE, “re”)] AND [LIMIT-TO (SRCTYPE, “j”)] AND (LIMIT-TO (LANGUAGE, “English”))]. This initial search yielded 1,581 publications. Following the PRISMA guidelines for identifying and screening relevant sources, as depicted in Figure 1, the results were meticulously screened (Moher et al., 2010). This involved limiting the scope to research articles and reviews published in English-language academic journals within the field of psychology, specifically between January 1st, 2003, and December 31st, 2023. To ensure the quality of the literature data, documents deemed irrelevant to the study’s topic were excluded. This included materials outside the field of psychology, non-English publications, book chapters, conference papers, books, notes, letters, and errata. This refined process resulted in the exclusion of 669 papers, leaving a final corpus of 912 documents for analysis. Comprehensive references and records from these documents were then exported in CSV format for further processing.

### 2.3 Data analysis

The study employed VOSviewer (1.6.17) and Microsoft Excel 2021 for analytics. Changes and patterns in the number of articles were examined using Microsoft Excel 2021, while document analysis was conducted using VOSviewer,^1^ a software configuration developed by Van Eck and Waltman. Widely recognized for its ability to produce excellent visualization and analytical results, VOSviewer is frequently used for document analysis. It offers a range of analytical techniques, including co-authorship and co-citation analysis, network and keyword analysis, density visualizations, clustering, and mapping and visualization techniques.

To better understand the current state of research on the psychological well‑being of children in foster care, the available literature was thoroughly reviewed using descriptive and bibliometric methods. This involved analyzing the number of publications and citations over time to track the growth of the field. We analyzed the field’s productivity by creating ranking tables for describing the core journals, research areas, leading authors, and countries that significantly contribute to the field’s advancement.

Bibliometric mapping, using the freely available software VOSviewer, visually represents the structure of a research field, encompassing social, intellectual, and conceptual aspects. In these maps, elements like journals, publications, or authors are depicted as circular nodes. Node size reflects volume (e.g., an author’s publication count), while node position indicates similarity to other nodes, with closer nodes being more alike. Nodes are visually linked to represent their connections, with line thickness reflecting the strength of these relationships. Each node’s color indicates its assigned cluster is determined by grouping nodes with similarities, providing a comprehensive visual representation of the field’s structure (Van Eck and Waltman, 2010).

To delve deeper into the research landscape’s structure, VOSviewer software utilized a distance-based approach involving a three-step process: normalizing differences between nodes, creating a two-dimensional map reflecting node similarity based on distance, and clustering closely related nodes (Van Eck and Waltman, 2011). Co-authorship analyses, focusing on authors and their affiliated countries/territories, were conducted to examine social structures within the research field. These analyses revealed collaborative networks and highlighted frequent co-publication patterns. Co-citation analysis of journals provided insights into the intellectual structure, with clusters representing disciplines underpinning the research field. Stronger co-citation relationships, indicated by frequent joint citations, suggested shared theoretical and semantic grounds. Lastly, co-occurrence analysis of author keywords unveiled the conceptual structure, with clusters representing prominent topical areas explored in the literature over the past two decades.

## 3 Results

### 3.1 Annual publication output analysis

**Figure 2:**
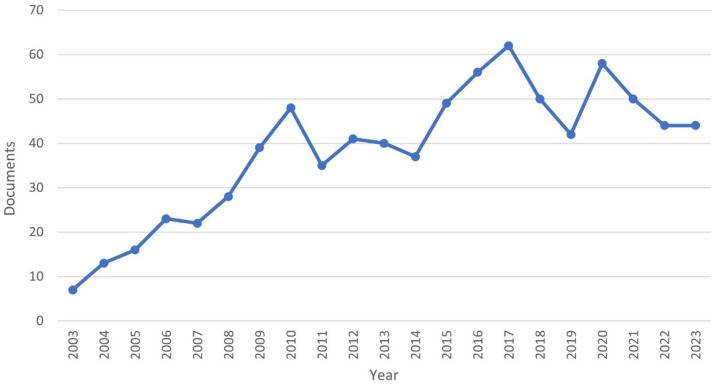
Yearly publications related to the study on the psychological well‑being of foster children from 2003 to 2023.

Analyzing annual publication output illuminates the research trends and growing importance of a particular field of study. Figure 2 presents the yearly publication numbers and their growth rate, drawn from the Scopus database, highlighting the development of research on the psychological well‑being of children in foster care. While the concept of psychological well‑being emerged earlier, dedicated research focusing on foster children remained limited in the early 21st century. Between 2003 (*n* = 7) and 2007 (*n* = 22), the number of publications remained relatively low. However, post-2007, there was a noticeable increase in research activity, reaching an initial peak in 2010 (*n* = 48). Despite a slight dip in 2011 (*n* = 35), publication numbers surged to their highest point in 2017, with a remarkable 62 articles published. Although a gradual decline followed in 2019, productivity has since stabilized, as evidenced by the consistent output in 2022 (*n* = 44) and 2023 (*n* = 44). This suggests a potential for continued growth in this research area in the coming years.

### 3.2 Most prolific countries/regions analysis

**Figure 3:**
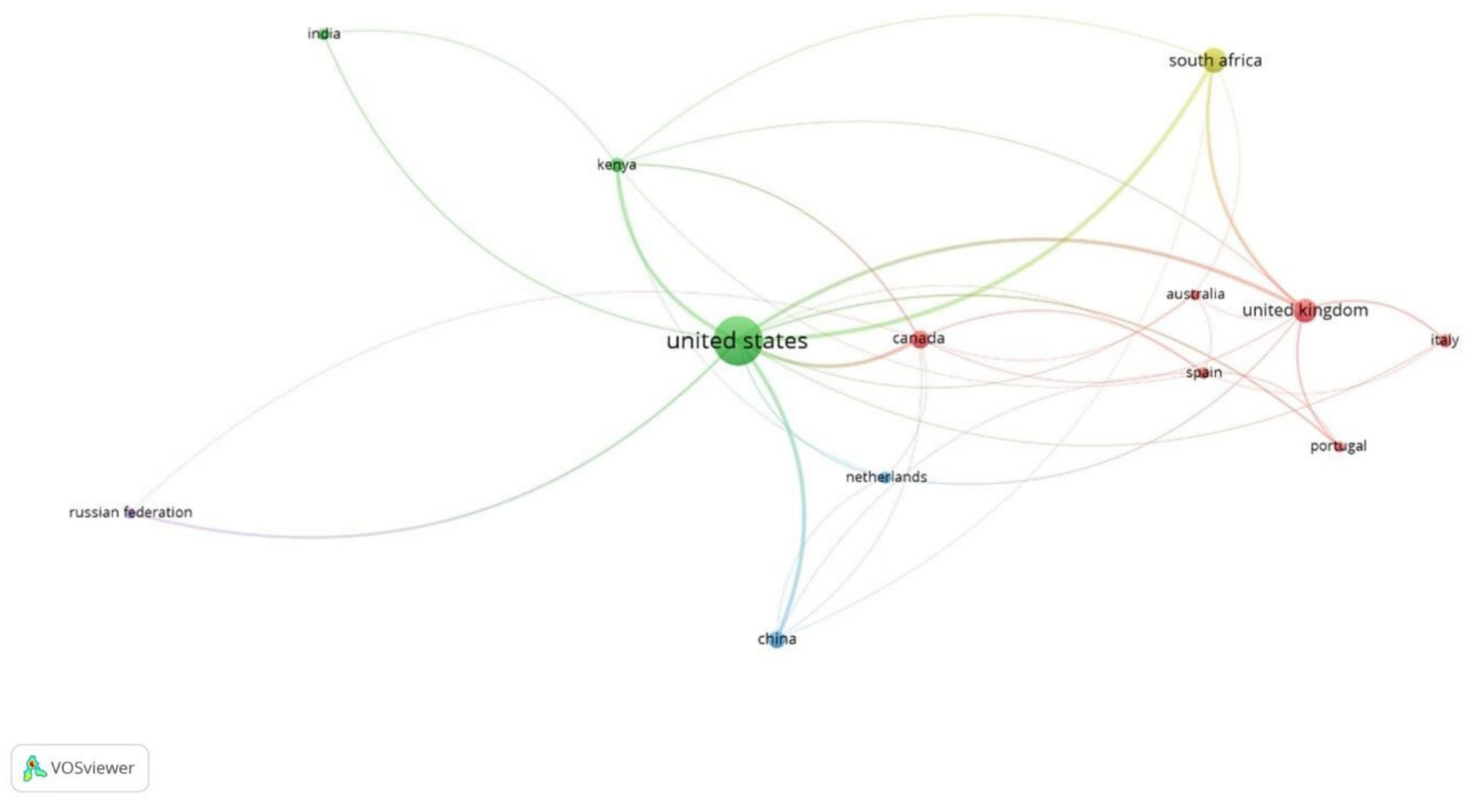
Mapping the top 10 countries/regions with a minimum of 20 publications.

Figure 3 illustrates the collaborative landscape of research on the psychological well‑being of children in foster care, highlighting countries/regions with multiple publications between 2003 and 2023. A total of 101 countries/regions contributed to this body of research. Using a threshold of at least 20 publications and a minimum link strength of 1, the network visualization comprises 13 nodes and 36 links, representing collaborating countries and regions. The United States emerges as the most prolific contributor, with 416 publications, constituting 45.61% (416/912) of the total output. Following the United States, the most active countries are South Africa (99), the United Kingdom (89), Canada (56), China (45), Kenya (34), Italy (23), the Netherlands (23), India (22), and Spain (22). Table 1 further ranks the top 10 contributing countries/regions based on overall link strength, Scopus citations, and the number of published articles. Notably, the United States holds the top position across all three metrics, underscoring its significant influence and expertise in this research domain.

**Table 1:** Summary of the top 10 most prolific countries/regions for the number of articles published.

Rank	Country/region	Publications	Frequency (%) *N* = 912	Scopus citations	Total link strength
1st	United States	416	45.61	12,105	276
2nd	South Africa	99	10.85	2,637	81
3rd	United Kingdom	89	9.75	3,220	112
4th	Canada	56	6.14	1,264	60
5th	China	45	4.93	834	46
6th	Kenya	34	3.72	670	48
7th	Italy	23	2.52	585	15
8th	Netherlands	23	2.52	794	16
9th	India	22	2.41	121	11
10th	Spain	22	2.41	348	22

### 3.3 Active organization analysis

**Figure 4:**
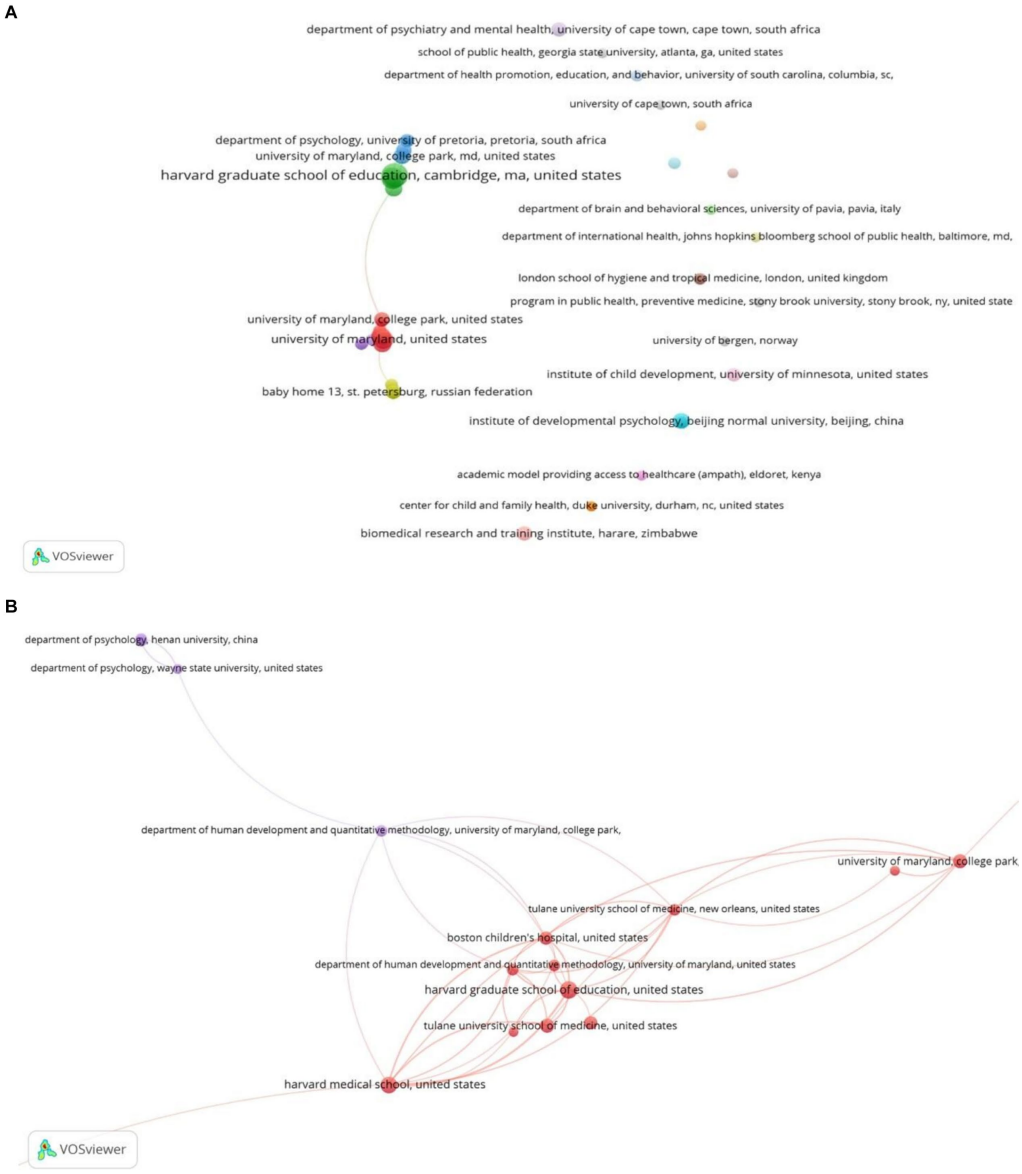
**(A)** Mapping of organizations in the study of psychological well‑being of foster children. **(B)** Mapping the top 10 prolific organizations with a minimum of 10 publications.

Figure 4, generated using VOSviewer software, visually identifies key contributing organizations, while Table 2 provides a ranked list of the top 10 most prolific organizations based on publication count (Tao et al., 2021). Harvard Medical School emerges as the leading contributor (*n* = 56, 6.14%), followed by the University of Maryland, College Park (*n* = 55, 6.03%), Boston Children’s Hospital (*n* = 47, 5.15%), Tulane University School of Medicine (*n* = 36, 3.94%), and Harvard Graduate School of Education (*n* = 32, 3.50%). Notably, eight out of the top 10 organizations are based in the United States, with the remaining two from China and South Africa. This highlights the substantial contribution of the United States to research on the psychological well‑being of children in foster care, particularly the significant role of Harvard Medical School.

**Table 2:** Summary of the top 10 prolific organizations contributed to the publications.

Rank	Organizations	Publications	Frequency (%) *N* = 912	Scopus citations	Total link strength	Country
1st	Harvard Medical School	56	6.14	331	26	United States
2nd	University of Maryland, College Park	55	6.03	730	21	United States
3rd	Boston Children’s Hospital	47	5.15	248	31	United States
4th	Tulane University School of Medicine	36	3.94	688	47	United States
5th	Harvard Graduate School of Education	32	3.50	439	68	United States
6th	Tulane University	31	3.39	513	7	United States
7th	Henan University	25	2.74	22	6	China
8th	Harvard University	21	2.30	160	27	United States
9th	Wayne State University School of Medicine	20	2.19	102	5	United States
10th	University of Cape Town	19	2.08	203	0	South Africa

### 3.4 Scholarly journals and co-cited journals analysis

**Figure 5:**
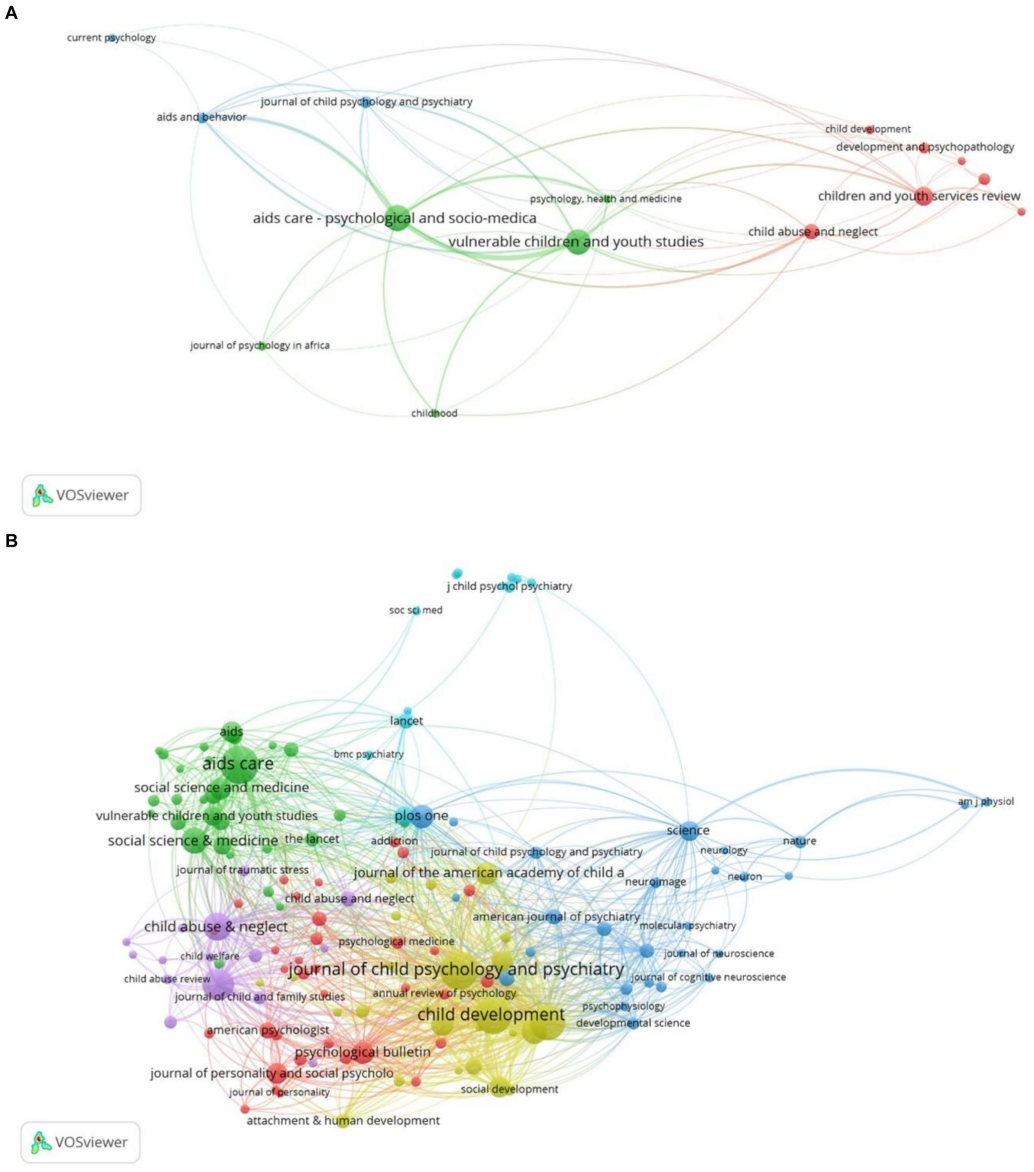
**(A)** Mapping the top 10 scholarly journals with a minimum of 10 publications. **(B)** Mapping of co-cited journals participating in the study of the psychological well‑being of foster children.

A total of 912 documents were published across 307 journals. Figure 5 presents the leading 10 scholarly journals in this field. *AIDS Care: Psychological and Socio-Medical Aspects of AIDS/HIV* emerges as the leading publication outlet with 91 documents, followed closely by *Vulnerable Children and Youth Studies* with 86 documents. *Children and Youth Services Review* published 47 documents, while *Child Abuse and Neglect* contributed 32 documents. *Frontiers in Psychology* ranks fifth with 19 published articles. These journals, along with others in the top 10, represent a multidisciplinary range, encompassing psychology, sociology, public health, social work, and anthropology. In 2022, most journals in this field had impact factors between 3 and 4, with the *Journal of Child Psychology and Psychiatry* being a notable exception with an impact factor of 7.6.

Table 3 also highlights the 10 most frequently cited journals, indicating their scholarly impact and prominence within the field. These journals have impact factors ranging from 1.7 to 13.3 (average IF: 5.17). The *Journal of the American Academy of Child and Adolescent Psychiatry* holds the highest impact factor (13.3), followed by the *Journal of Child Psychology and Psychiatry* (7.6) and *Social Sciences and Medicine* (5.4).

**Table 3:** An overview of the articles in the top 10 scholarly journals and co-cited journals.

Rank	Publications	IF (2022)	Journal	Co-citation	IF (2022)	Co-cited journals
1st	91	1.7	AIDS Care—Psychological and Socio-Medical Aspects of AIDS/HIV	630	7.6	Journal of Child Psychology and Psychiatry
2nd	86	1.1	Vulnerable Children & Youth Studies	629	1.7	Aids Care—Psychological and Socio-Medical Aspects of AIDS/HIV
3rd	47	3.3	Children and Youth Services Review	610	4.6	Child Development
4th	32	4.8	Child Abuse and Neglect	548	3.3	Development and Psychopathology
5th	19	3.8	Frontiers in Psychology	449	3.3	Children and Youth Services Review
6th	19	7.6	Journal of Child Psychology and Psychiatry	335	4.8	Child Abuse and Neglect
7th	17	4.4	AIDS and Behavior	296	5.4	Social Sciences & Medicine
8th	15	3.3	Development and Psychopathology	279	4.0	Developmental Psychology
9th	12	1.2	Journal of Psychology in Africa	240	3.7	PLoS ONE
10th	12	4.6	Child Development	227	13.3	Journal of the American Academy of Child and Adolescent Psychiatry

In terms of co-citation counts, the *Journal of Child Psychology and Psychiatry* leads with 630 co-citations, followed by *AIDS Care: Psychological and Socio-Medical Aspects of HIV/AIDS* (629), *Child Development* (610), and *Development and Psychopathology* (548). This co-citation analysis identifies the *Journal of Child Psychology and Psychiatry* as a central publication in the study of psychological well‑being among children in foster care homes.

### 3.5 Productive authors and co-cited authors’ analysis

**Figure 6:**
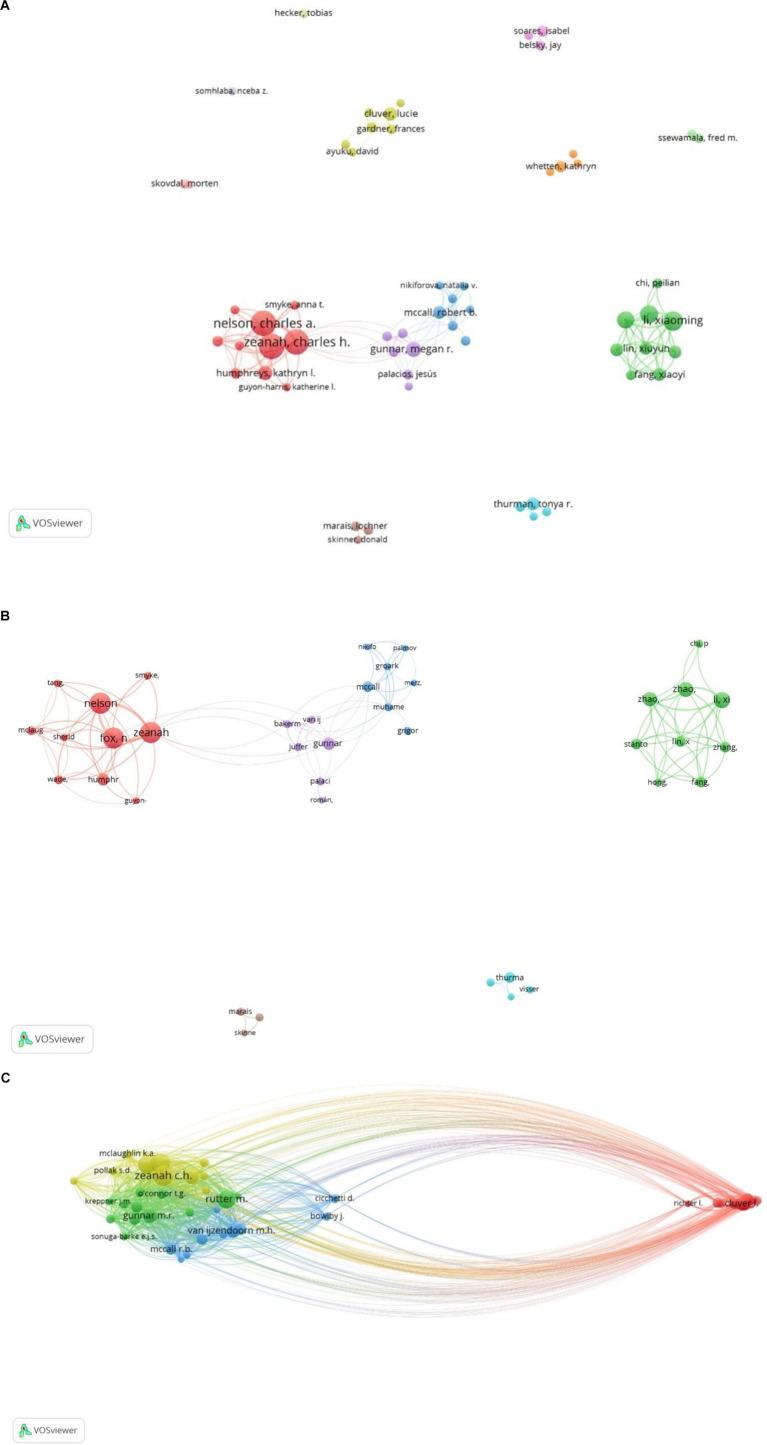
**(A)** Mapping of main authors in the field of psychological well‑being of foster children. **(B)** Mapping of the largest collaboration network of top 10 authors. **(C)** Mapping of co-cited authors engaging in the study of the psychological well‑being of foster children.

Figure 6A visualizes the network of authors who have published more than 10 articles in the field, generated using VOSviewer. The resulting map comprises 60 nodes (authors) and 134 links (collaborations). Figure 6B further highlights the most extensive collaboration network, which includes prominent authors such as Nelson, C.A., Zeanah, C.H., Fox, N.A., Li, X., and Thurman, T.R. In terms of publication productivity, Nelson, C.A. leads with 52 research articles, followed by Zeanah, C.H. (50 articles) and Fox, N.A. However, when considering citations, Zeanah, C.H. emerges as the most influential author with 2,417 citations, followed by Nelson, C.A. (2,211 citations) and Fox, N.A. (2,105 citations).

Table 4 provides a consolidated view of the top 10 authors based on both publication count and citation impact, highlighting their prolific contributions and influence within the field. Furthermore, the analysis identifies the 10 most frequently co-cited authors, indicating a strong influence on the shared knowledge base of the field. Zeanah, C.H. (876 co-citations) emerges as the most frequently co-cited author, followed by Nelson, C.A. (836 co-citations), Fox, N.A. (711 co-citations), and Rutter, M. (657 co-citations). These authors have made substantial contributions to understanding the psychological well‑being of foster children in residential care settings.

**Table 4:** Summary of the topmost 10 productive authors and co-authors in the study of psychological well‑being of foster children.

Rank	Publications	Authors	Citations	Total link strength	Co-cited Authors	Co-citations
1^st^	52	Nelson, C. A.	2,211	146	Zeanah, C. H.	876
2^nd^	50	Zeanah, C. H.	2,417	151	Nelson, C. A.	836
3^rd^	49	Fox, N.A.	2,105	147	Fox, N. A.	711
4^th^	26	Zhao, J.	460	104	Rutter, M.	657
5^th^	25	Li, X.	620	111	Smyke, A. T.	426
6^th^	22	Zhao, G.	474	105	Gunnar, M. R.	420
7^th^	17	Gunnar, M. R.	838	11	Van-ijzendoorn, M. H.	397
8^th^	16	Thurman, T. R.	306	11	Bakermans-kranenburg, M. J.	275
9^th^	15	Fang, X.	388	78	Juffer, F.	266
10^th^	15	Humphreys, K. L.	309	47	Beckett, C.	257

### 3.6 Cited references analysis

Table 5 showcasing the top 10 most cited references in the field, offers valuable insights into the foundational research on the psychological well‑being of children in foster care. This analysis helps illuminate connections between organizations, countries, and authors, highlighting key themes and influential works in the field.

**Table 5:** An overview of the 10 most-cited references.

Rank	Citation	Year	Author(s)	Title	Source	Country
1st	370	2005	Zeanah, C. H.	Attachment in Institutionalized and Community Children in Romania (Zeanah et al., 2005)	Child Development	United States
2nd	351	2004	Case, A.	Orphans in Africa: Parental Death, Poverty, and School Enrolment (Case et al., 2004)	Demography	United States
3rd	312	2007	Cluver, L.	Psychological Distress amongst AIDS-Orphaned Children in Urban South Africa (Cluver et al., 2007)	Journal of child psychology and psychiatry	United Kingdom
4th	197	2002	Makame, V.	Psychological Well-being of Orphans in Dar El Salaam, Tanzania (Makame et al., 2002)	Acta Paediatrica	United Kingdom
5th	155	2008	Bruskas, D.	Children in Foster Care: A Vulnerable Population at Risk (Bruskas, 2008)	Journal of Child and Adolescent Psychiatric Nursing	United States
6th	84	2007	Bhargava, A.	AIDS Epidemic and the Psychological Well-being and School Participation of Ethiopian Orphans (Bhargava, 2005)	Psychology, Health & Medicine	United States
7th	29	2011	Okawa, S.	Perceived Social Support and the Psychological Well-being of AIDS Orphans in Urban Kenya (Okawa et al., 2011)	AIDS Care—Psychological and Socio-Medical Aspects of AIDS/HIV	Japan
8th	24	2014	Salifu, Y. J.	Stress, Coping and Quality of Life: An Exploratory Study of the Psychological Well-being of Ghanaian Orphans Placed in Orphanages (Salifu Yendork and Somhlaba, 2014)	Children and Youth Services Review	Ghana
9th	21	2020	Ntuli, B.	The Psychosocial Well-being of Orphans: The Case of Early School Leavers in Socially Depressed Environment in Mpumalanga Province, South Africa (Ntuli et al., 2020)	PLoS ONE	South Africa
10th	17	2018	Williams-Butler, A.	Relational Permanence and Psychological Well-being among African American Adolescents in Foster Care (Williams-Butler et al., 2018)	Journal of Child and Family Studies	United States

**Figure 7:**
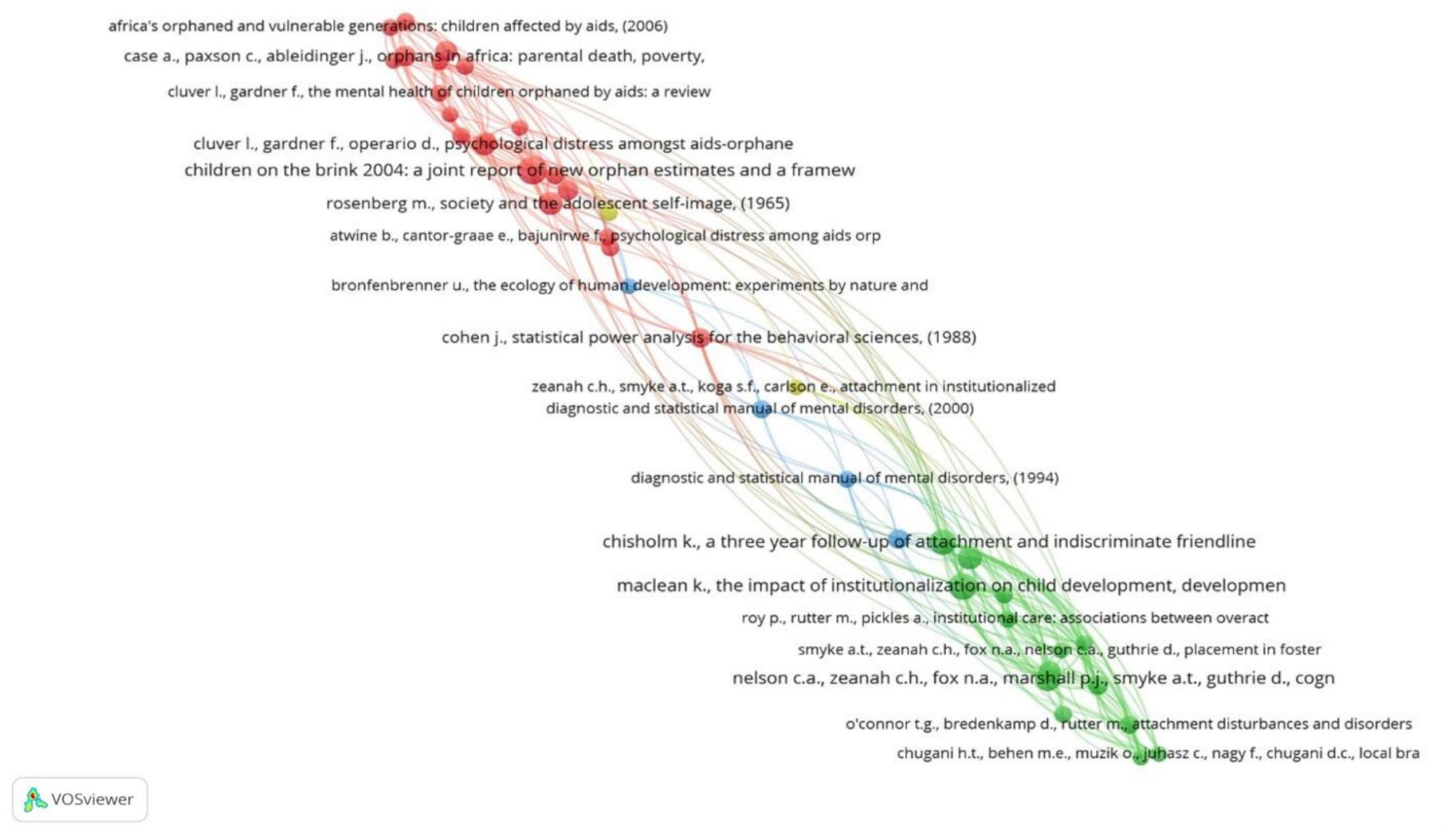
Mapping of reference citation network analysis.

Figure 7 presents a visualization of the citation network generated using VOSviewer software. The most cited reference, “Attachment in Institutionalized and Community Children in Romania” by Zeanah et al. (2005), underscores the significance of attachment research in understanding the experiences of children in foster care. This seminal work, published in the *Journal of Child Development* in 2005, highlights the journal’s influence and the enduring relevance of attachment theory in this research area. Case et al. (2004) with their work, “Orphans in Africa: Parental Death, Poverty, and School Enrolment,” ranks second, emphasizing the intersection of socioeconomic factors and well‑being for children in vulnerable circumstances. Cluver et al. (2007) further underscore the vulnerability of this population with their study “Psychological Distress amongst AIDS-Orphaned Children in Urban South Africa,” focusing on the psychological impact of AIDS on children. Similarly, Makame et al. (2002) contribute important findings from the Tanzanian context with their research “Psychological Well-being of Orphans in Dar El Salaam, Tanzania,” further highlighting the diverse experiences of orphans globally. Bruskas (2008), in their study “Children in Foster Care: A Vulnerable Population at Risk,” directly address the challenges faced by foster children, emphasizing their heightened vulnerability. The prominence of these works, alongside others in the top 10, underscores the critical focus on psychological well‑being, quality of life, and the experiences of orphans and foster children across different geographical contexts.

The dominance of US-based authors like Zeanah, Case, Bruskas, Bhargava, and Williams-Butler in the top 10 most cited works suggests a significant influence of the United States in shaping research on the psychological well‑being of children in foster care settings.

### 3.7 Co-occurrence of keywords analysis

Keywords are essential elements in academic literature as they offer a concise summary of a research topic and effectively convey the core content of an article. By analyzing keywords, we can gain insights into the central themes and emerging trends within a specific field. One effective method is keyword co-occurrence analysis, which involves calculating how frequently pairs of keywords appear together within the same research papers. this approach allows researchers to identify research hotspots by examining the composition, distribution, and clustering of keywords. For instance, a study by Zhu et al. (2022) demonstrated how analyzing keyword co-occurrence can effectively uncover the distribution of research hotspots within a specific field.

**Figure 8:**
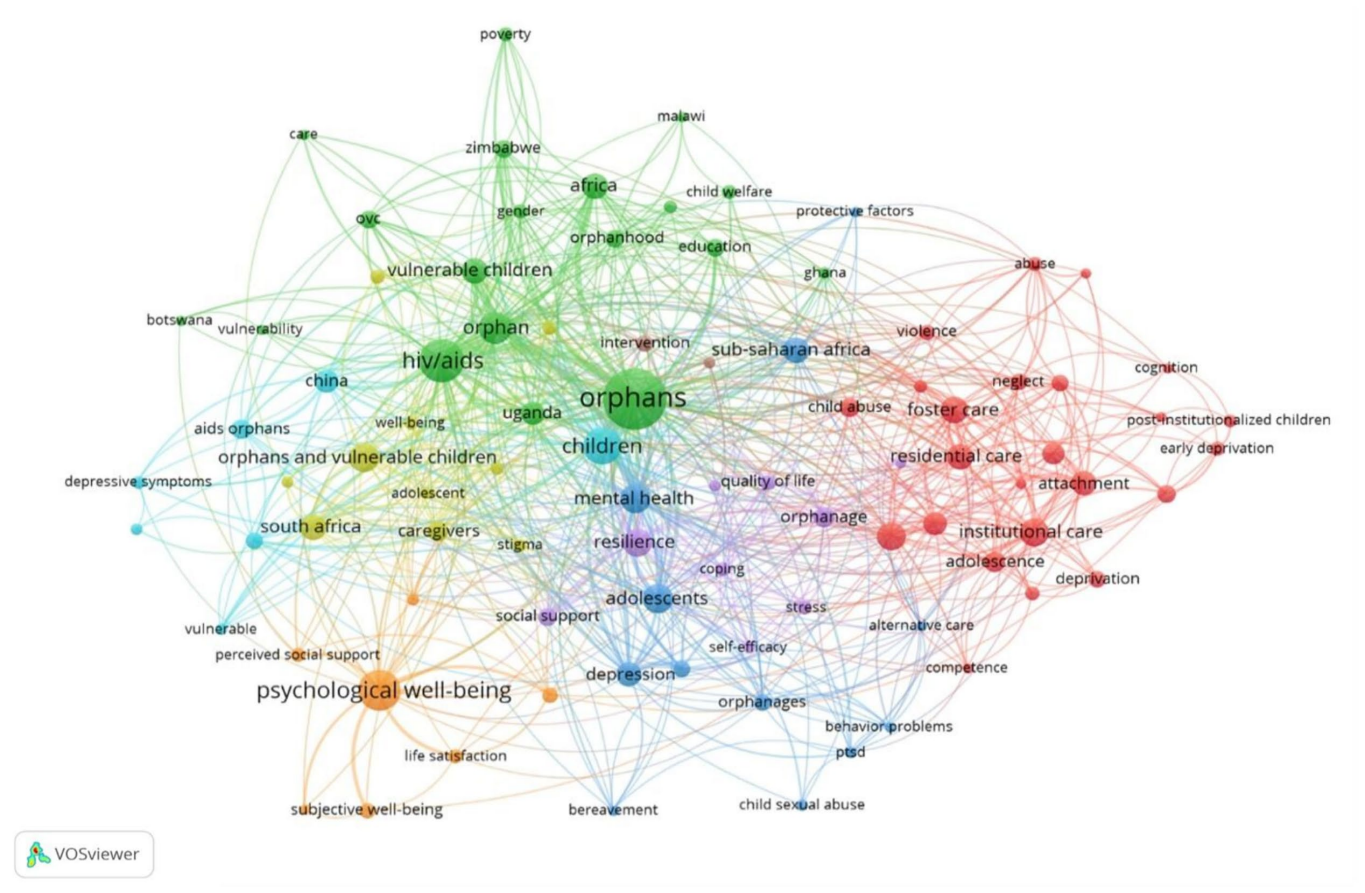
Mapping of keyword co-occurrence network related to the psychological well‑being of foster children.

Figure 8 provides a visual representation of the cooperative network for keyword co-occurrences. In this particular study the search terms included “psychological well‑being,” “children,” “adolescents,” “foster care homes,” “residential care,” “institutionalized children,” “orphan,” and “semi-orphan.” notably the keyword “HIV/AIDS orphans” emerged as a significant hub exhibiting strong connections with other keywords.

Table 6 presents a ranked list of the top 30 keywords based on their frequencies and co-occurrences. The analysis highlights several terms that have garnered considerable attention in research on the psychological well‑being of children in foster care. The most frequently used keywords, representing key areas of research interest, include: “orphans” (157), “AIDS/HIV orphans” (78), “psychological well‑being” (70), “orphans and vulnerable children” (37), “mental health” (35), “institutionalization” (33), “institutional care” (33), “resilience” (32), “foster care” (29), “residential care” (28) and “orphanages” (17). The prominence of these keywords, as evidenced by their large nodes in the co-occurrence network, highlights their significance within the field.

**Table 6:** Summary of the top 30 keywords for the psychological well‑being of foster children based on frequency.

Rank	Frequency	Keyword	Rank	Frequency	Keyword
1	157	Orphans	16	24	Depression
2	78	HIV/AIDS orphans	17	21	Adoption
3	70	Psychological well‑being	18	19	Institutionalized children
4	58	Children	19	18	Adolescence
5	42	Orphan	20	17	Orphanage
6	37	Orphans & Vulnerable Children	21	17	Caregivers
7	36	Adolescents	22	12	Intervention
8	35	Mental health	23	11	Anxiety
9	33	Institutional care	24	10	Quality of life
10	33	Institutionalization	25	10	Self-esteem
11	32	Resilience	26	10	Subjective well‑being
12	29	Foster care	27	9	Life satisfaction
13	28	Residential care	28	8	Perceived social support
14	26	Vulnerable children	29	6	Self-efficacy
15	24	Attachment	30	6	Psychosocial well‑being

### 3.8 Hot research topics

Analyzing keywords is essential for summarizing study topics and exploring research hotspots within a specific field. Examining keyword frequency in the study field and specialized disciplines can provide a comprehensive overview of the research on the psychological well‑being of children in foster care. The size of nodes denotes keyword frequency, while edge thickness represents the level of keyword co-occurrence (Hernández-Torrano et al., 2020). Five distinct clusters emerge from the analysis:

*Cluster 1 (Red)*: This cluster examines the concept of *“institutional care”* as a prominent model within child welfare services, encompassing various settings where children receive care outside of a traditional family setting. The term “institutional care” emerges as the largest node, reflecting its significance in this area. It connects to 29 keywords, including “residential care,” “foster care,” and “orphanages,” highlighting different forms and aspects of foster care homes.
*Cluster 2 (Green)*: This cluster focuses on factors related to orphans, who often fall under the care of foster systems. The term *“orphans”* appears as the largest node, reflecting its significance in this area of research. It connects to 67 keywords, encompassing a broad range of topics relevant to the experiences of orphans, including “vulnerable children,” “HIV/AIDS,” “poverty,” “care,” “gender,” and “education.”
*Cluster 3 (Blue)*: This cluster focuses on the *mental health concerns* of children, particularly those in foster care. *The* central node, *“children,”* underscores the cluster’s emphasis on the well‑being of this vulnerable group. This central node connects to 47 keywords that represent a spectrum of emotional and behavioral challenges relevant to foster children. These keywords include “adolescents,” “mental health,” “depression,” “behavioral problems,” “post-traumatic stress disorder,” “child sexual abuse,” “AIDS orphans,” “depressive symptoms,” and “anxiety.”
*Cluster 4 (Orange)*: This cluster centers on the concept of *“psychological well‑being,”* represented by the largest node, *and* explores factors that contribute to positive mental health in foster children. The 32 keywords associated with this cluster include “well‑being,” “caregiver,” “life satisfaction,” “subjective well‑being,” “perceived social support,” “self-esteem,” and “psychosocial well‑being.”
*Cluster 5 (Purple)*: This cluster delves into the crucial role of *coping strategies* in fostering psychological well‑being, particularly for children facing challenging circumstances. Keywords within this cluster include “resilience,” “quality of life,” “social support,” “coping,” “self-efficacy,” “stress,” and “intervention,” highlighting both internal resources and external support systems that contribute to positive adaptation.

## 4 Discussion

This bibliometric analysis examined research published between 2003 and 2023 on the psychological well‑being of children in foster care homes using VOSviewer software. The yearly publication trends, influential countries, key journals, active organizations, and productive authors were analyzed to provide insights for future research. Initially, research in this area was limited, with publications increasing gradually after 2003 and significantly after 2010. While there were fluctuations, the field stabilized in recent years, suggesting continued growth. The United States has emerged as a leading contributor to this field, evidenced by its high publication output and the dominance of US institutions among the top 10 organizations. China and South Africa also demonstrate considerable research engagement. The following journals are identified as crucial publication platforms: *AIDS Care—Psychological and Socio-Medical Aspects of AIDS/HIV, Vulnerable Children and Youth Studies*, *Children and Youth Services Review, Child Abuse and Neglect,* and *Frontiers in Psychology*. Researchers can also benefit from examining frequently cited journals like the *Journal of Child Psychology and Psychiatry*, *AIDS Care: Psychological and Socio-Medical Aspects of AIDS/HIV*, *Child Development*, *Development and Psychopathology*, and *Children and Youth Services Review*. The analysis identified a prominent network of collaborating authors, including Nelson, C.A., Zeanah, C.H., and Fox, N.A., who have made substantial contributions to the field through their prolific publication and citation records. Their work represents a valuable resource for understanding the psychological well‑being of children in foster care.

## 5 Recent trends

Beyond foundational knowledge of prominent research topics, a comprehensive analysis of research trends is essential to identify emerging fields and predict future research frontiers. Research on psychological well‑being has made significant strides, revealing its multifaceted nature and impact on foster children’s lives. A key area of focus within this field is the psychological well‑being of children in foster care, particularly orphans. This research analysis delves into the trends shaping our understanding of the challenges and support mechanisms related to the psychological well‑being of orphans.

Studies consistently demonstrate the vulnerability of orphaned children in institutional settings, highlighting the detrimental effects on their psychological well‑being. Children raised in institutions often exhibit a higher prevalence of internalizing and externalizing behaviors, and lower well‑being than their non-institutionalized peers due to encountering adverse experiences throughout their childhood (Bruskas and Tessin, 2013; Padmaja et al., 2014). This disparity is attributed to various factors, including the stressful nature of institutional environments, limited opportunities for emotional support, and the potential for developmental disruptions (Yimer, 2022). Early experiences of loss, resource scarcity, and disrupted attachments contribute significantly to the emotional and behavioral challenges faced by orphans (Gudyanga et al., 2015). Furthermore, research suggests that the type of orphanhood experienced can influence specific dimensions of well‑being. For instance, while both half- and double-orphans may face challenges, half-orphans tend to report higher levels of self-acceptance and positive relationships, indicating potential protective factors associated with having one surviving parent (Malla et al., 2019).

This heightened vulnerability to adverse experiences within institutional settings has significant implications for the mental health of orphaned children and adolescents. A prominent research trend centers on their increased susceptibility to mental health issues. Studies highlight a prevalence of behavioral and emotional problems, including conduct disorders, peer difficulties, emotional dysregulation, hyperactivity, and underdeveloped prosocial skills (Rahman et al., 2012; Kaur et al., 2018; Shiferaw et al., 2018). This vulnerability stems from the traumatic experiences associated with orphanhoods, such as the loss of parents, potential exposure to neglect or abuse, and the instability of institutional environments. Adding to these immediate challenges, research further explores the long-term impact of orphanhood on mental health. Findings indicate a higher susceptibility to conditions such as anxiety, depression, stress, and social dysfunction (Dey and Daliya, 2019). Parental deprivation emerges as a significant factor influencing mental health outcomes (Kumari and Jahan, 2020), with studies consistently reporting elevated levels of depression, loneliness, post-traumatic stress disorder, and limited social support among individuals who have experienced the loss of parents (Zhao et al., 2010; Shiferaw et al., 2018; Bhatt et al., 2020).

Given the profound impact of orphanhood on mental health, researchers are increasingly exploring interventions and support systems to enhance the well‑being of this vulnerable population. A growing body of literature emphasizes the importance of psychosocial support services tailored to the specific needs of orphaned and vulnerable children (Bimha and Sibiya, 2023). This includes providing access to counseling services, training caregivers and educators in psychosocial support strategies, and creating supportive environments that foster resilience and emotional regulation (Khalid et al., 2023). Beyond these immediate support mechanisms, recent research highlights the long-term benefits of enhancing caregiving quality in foster homes, suggesting that such interventions can positively impact children’s social skills, executive functions, and overall development (Zeytinoglu et al., 2023).

Despite their vulnerability, orphans often demonstrate a remarkable capacity for resilience. While they may exhibit lower self-esteem and quality of life, studies suggest that orphaned children may possess a greater resilience capacity than their non-orphaned counterparts (Salifu Yendork and Somhlaba, 2014; Erango and Ayka, 2015; Katyal, 2015). This resilience is particularly evident in children in foster care, who often demonstrate remarkable resilience, likely developed through coping mechanisms honed in the face of adversity. By understanding the factors that contribute to this resilience, we can develop interventions to further bolster their ability to thrive despite early life challenges. Further amplifying the importance of resilience, emerging research emphasizes the role of resilience and emotional regulation in fostering psychological well‑being among adolescents, highlighting potential avenues for intervention and support (Hamidi et al., 2023). These emerging trends underscore the urgent need for targeted interventions and robust support systems designed to address the psychological and emotional needs of children in foster care. By fostering resilience, we can pave the way for improved well‑being and positive long-term outcomes.

## 6 Future trends

Future trends highlight the urgent need to bolster intervention programs for orphans and foster children beyond addressing basic needs. There is a growing emphasis on incorporating emotional support, counseling services, and training for caregivers and educators. Furthermore, empowering children in foster care with essential life skills interventions, such as stress management, resilience-building, problem-solving, and decision-making, is gaining traction (Hailegiorgis et al., 2018; Shafiq et al., 2020; Duraisamy et al., 2023a,b). This shift towards a more holistic and therapeutic approach signifies a promising direction for future research and program development. To enhance the psychological well‑being of these vulnerable populations, researchers (Cherewick et al., 2023) suggest prioritizing interventions that foster strong community ties, autonomy, and self-esteem. However, further research is needed to explore the factors that affect autonomy and self-acceptance within these populations. Looking ahead, it is crucial to evaluate the long-term effectiveness of psychosocial interventions and conduct longitudinal studies to gain a deeper understanding of the experiences of adolescent orphans. Additionally, there is a pressing need to raise awareness and provide rehabilitative programs specifically designed for orphans. These programs should focus on developing emotional intelligence, self-esteem, and other positive psychological qualities that are essential for their future success. Furthermore, developing targeted psychological intervention strategies that address the unique cognitive, emotional, and behavioral needs of specific orphan groups is paramount. By pursuing these future directions, researchers and practitioners can contribute to improved well‑being and brighter futures for orphans and foster children.

## 7 Conclusion

This bibliometric analysis maps research trends on the psychological well‑being of children in foster care, spanning from 2003 to 2023. Findings reveal that addressing emotional and behavioral issues, alongside understanding the unique needs of children in residential care, are crucial for improving their overall well‑being. This study underscores the critical need for developing effective interventions to support these children. Furthermore, it highlights the importance of holistic approaches that not only address basic needs but also prioritize emotional support. By integrating these strategies, we can empower these vulnerable children to thrive despite the challenges they face.

The significance of this study lies in its use of bibliometric analysis to shed light on the evolving landscape of research concerning the psychological well‑being of children in foster care. By visually mapping research trends, we gain a deeper understanding of the field’s trajectory, enabling us to pinpoint promising avenues for future exploration more effectively. Our analysis underscores the critical importance of psychological well‑being for these children, emphasizing its connection to the development of intervention programs aimed at fostering positive life outcomes. Our findings reveal prominent research clusters centered around the impact of well‑being on children in institutional care, mental health concerns, and the significance of psychosocial interventions and resilience-building strategies. Notably, a thorough review of existing research revealed no prior bibliometric studies examining scholarly output on the psychological well‑being of children specifically in foster care homes. This gap in the literature highlights the valuable opportunity presented by the present study to provide insights into the current state of global research on this important topic. Our findings illuminate existing gaps in knowledge and pave the way for further research on interventions and support systems tailored to enhance the well‑being of foster children. By highlighting these crucial aspects, this bibliometric study offers valuable insights for researchers, policymakers, and child welfare professionals working to improve the lives of vulnerable children in foster care.

## 8 Strengths and limitations

This study offers a comprehensive and insightful exploration of research trends on the psychological well‑being of children in foster care. By employing rigorous bibliometric methodologies and a substantial dataset from the Scopus database, the study effectively maps the evolution of research in this crucial area, providing an up-to-date and granular examination of publication trends, hot research topics, influential authors, and leading institutions, all contributing to a deeper understanding of the field. Notably, a significant research gap identified by the study is the scarcity of bibliometric studies specifically focused on the psychological well‑being of children in foster care homes, making this contribution a valuable foundation for future research. The analysis, enhanced by VOSviewer visualizations of research networks, offers a unique perspective on the intellectual structure of the field, enabling the identification of emerging research frontiers. Through systematic data analysis, the study not only contributes significantly to understanding the current state of knowledge but also illuminates promising avenues for future investigation in this domain. Despite its strengths, the study’s reliance on the Scopus database may have resulted in the exclusion of relevant studies indexed elsewhere. Additionally, focusing on English-language publications could have limited the scope of the analysis, and the use of keyword analysis may have inadvertently omitted research employing different terminology.

## 9 Recommendation

To provide a more comprehensive understanding of the field, future research should address these limitations by expanding data sources, search parameters, and language inclusion. Incorporating alternative methodologies like meta-analysis can provide a more comprehensive understanding of the research field. Encouraging researchers to collaborate with a wider network of peers and organizations can foster a more interconnected research landscape, potentially leading to more impactful and generalizable findings.

## Data Availability

The original contributions presented in the study are included in the article/supplementary material, further inquiries can be directed to the corresponding author.

## Footnotes

^1^ www.vosviewer.com
